# Gait and balance dysfunction are associated with cognitive performance only in men with Parkinson’s disease

**DOI:** 10.1016/j.prdoa.2025.100363

**Published:** 2025-07-05

**Authors:** Amy W. Amara, Kimberly H. Wood, Aya M. Miften, Lina Kleinschmidt, Corey S. White, Allen Joop, Raima A. Memon, Jennifer Pilkington, Jutaluk Kongsuk, Corina Catiul, Adeel A. Memon, Marcas M. Bamman, Christopher P. Hurt

**Affiliations:** a Department of Neurology, University of Colorado, Anschutz Medical Campus, Aurora, CO 80045, USA; b Department of Neurology, University of Alabama at Birmingham, Birmingham, AL 35294, USA; c UAB Center for Exercise Medicine, Birmingham, AL 35205, USA; d Department of Psychology, Samford University, Birmingham, AL 35229, USA; e Department of Cell, Developmental, and Integrative Biology, University of Alabama at Birmingham, Birmingham, AL 35294, USA; f Department of Psychology, University of Louisiana at Monroe, Monroe, LA 71209, USA; g Department of Pathology, Roswell Park Comprehensive Cancer Center, Buffalo, NY 14203, USA; h Department of Physical Therapy, University of Alabama at Birmingham, Birmingham, AL 35233, USA; i Department of Physical Therapy, Faculty of Allied Health Sciences, Naresuan University, Phitsanulok, Thailand; j Department of Neurology, West Virginia University Rockefeller Neuroscience Institute, Morgantown, WV 25605, USA; k Florida Institute for Human & Machine Cognition, Pensacola, FL 32502, USA

## Abstract

**Introduction:**

Parkinson’s disease (PD) can cause locomotor dysfunction and cognitive impairment and these symptoms are often associated. This study assesses relationships between comprehensive cognitive performance and locomotor function domains: gait, balance, and mobility, in men and women with PD.

**Methods:**

Sixty PD participants, Hoehn and Yahr 1–3, age ≥ 45, and Montreal Cognitive Assessment score ≥ 18, underwent locomotor function assessments and a comprehensive neurocognitive battery. Primary outcomes included Timed Up and Go (TUG), maximum walking speed (MWS), Mini Balance Evaluation Systems Test (Mini-BESTest), and cognitive composite score (CCS). Spearman correlations determined associations between cognition and locomotor function for the total sample and separately for men and women.

**Results:**

CCS correlated with all primary locomotor outcomes for the overall group and men only. Specifically, positive relationships were observed between the CCS and Mini-BEST Test (balance) for the overall group (p = 0.009) and men (p = 0.001), as well as MWS (gait) for the overall group (p = 0.006) and men (p < 0.001). CCS and TUG (mobility) were negatively correlated for the overall group (p = 0.005) and men (p < 0.001). There were no significant correlations between CCS and locomotor outcomes in women. Exploratory analyses suggest that performance in executive function, visuospatial skills, and processing speed drive these relationships.

**Conclusion:**

In PD, balance, gait, and mobility correlated with global cognition. Correlations between motor function and cognitive performance were evident only in men. This study enhances understanding of the complex interplay between cognition and motor function in PD, especially among men.

## Highlights

• Balance, gait, and mobility are correlated with cognition in Parkinson’s Disease.
• Correlations between cognition and locomotion are present only in men.
• Cognitive impairment may indicate gait, mobility, and balance dysfunction in men.
• Multiple cognitive domains are related to locomotor function in Parkinson’s disease.

Parkinson’s disease (PD) is characterized by progressive dopaminergic neurodegeneration in the substantia nigra pars compacta, causing observable dysfunction in motor systems responsible for gait, balance, and mobility [[1], [2], [3]]. Persons with Parkinson’s (PwP) therefore often have difficulties walking or performing other motor tasks, which impedes general mobility and increases risk of falls [4]. Further, up to 80% of PwP experience deficits in global cognition or individual cognitive domains [5]. Cognitive dysfunction can directly interfere with daily activities and impair quality of life, as well as increase caregiver burden and risk of institutionalization [[6], [7], [8]].

Work investigating gait, balance, and mobility in PwP has identified associations between locomotor function and cognitive performance [1,2]. Specifically, PwP with diminished cognitive function are more likely to display phenotypic postural instability/gait disorder (PIGD) [9], and patients with balance impairment are more likely to experience progressive cognitive decline [10]. Further, individuals who present with gait difficulty earlier in the disease process tend to develop the PIGD phenotype and develop early cognitive dysfunction as well [11]. While these studies demonstrate associations between cognition and motor function in PD, there is a gap in evaluating comprehensive cognitive function and directly linking it to gait, balance, and mobility. Understanding how specific cognitive deficits impact these physical domains can guide more targeted interventions.

Prior work has identified relationships between individual cognitive domains and gait/balance in PD. Specifically, slower walking speed and turning appear to be related to executive function, whereas balance control appears more related to visuospatial function [12]. Specific measures of visuospatial function, including response accuracy, discrimination threshold, and vestibular perception sensitivity also appear to be correlated with worsened gait and balance in PwP [13]. Further, executive function impairment may be associated with more global motor deficits including the postural instability/gait disorder phenotype, while visuospatial impairment and memory impairment may have more specific associations with freezing of gait and postural instability, respectively [9].

Differences in locomotion and cognition exist between men and women with PD. For example, differences observed for step width and step length while walking [14] have been attributed to greater ankle flexion through stance for women compared to men resulting in less plantarflexion at footoff [15]. In addition, men with PD appear to have greater impairment in global cognition, processing speed, verbal recall, and verbal fluency, whereas women with PD exhibit greater impairment in visuospatial functioning [16].

Despite evidence supporting relationships in PD between locomotion and cognition, locomotion and sex, and sex and cognition, to our knowledge, no work has been done to comprehensively investigate the interplay between these three variables in PwP. Therefore, the purpose of this study was to assess relationships between different locomotor function domains – specifically gait, balance, and mobility – and comprehensive cognitive performance in PwP. We hypothesized that impaired locomotor function would be associated with impaired cognitive performance. Additionally, we investigated differences in these relationships between men and women with PD.

## 1 Methods

### 1.1 Participants

This cross-sectional, observational study investigated relationships between cognitive performance and locomotor function domains, specifically gait, balance, and mobility, in PwP. Sixty participants with idiopathic PD (based on MDS diagnostic criteria: presence of bradykinesia as well as rest tremor and/or rigidity [17]) participated. Fifty-seven participants (95%) had clinically established PD and 3 participants (5%) had clinically probably PD (2 had red flags of falls within 3 years of diagnosis and 1 had red flag of bilateral symmetric parkinsonism, all 3 with at least one supportive criterion). PD participants, Hoehn and Yahr stage 1–3, and age ≥ 45, were recruited from the University of Alabama at Birmingham (UAB) Movement Disorders Center. Additional inclusion criteria included Montreal Cognitive Assessment (MoCA) score ≥ 18 and stable medications for at least four weeks prior to enrollment. Potential participants were excluded if they had deep brain stimulation. The UAB Institutional Review Board approved the study and all participants agreed to participate through written informed consent.

### 1.2 Assessments

#### 1.2.1 Motor function

This study assessed multiple domains of locomotor function to comprehensively assess movement capacity. The Timed Up and Go (TUG) test [18], in which individuals stand up from a chair, walk 3 m, turn around, walk back to the chair, and sit down, was used to assess mobility. Maximum walking speed (MWS) was used to evaluate gait function. Iindividuals completed 3 walking trials on an overground 10 m walkway where we timed the middle 6 m to allow for acceleration and deceleration to their steady-state speed. Individuals were instructed to “walk at the fastest speed you feel safe”. Participants also performed a clinical assessment of mobility and balance, the Mini Balance Evaluation Systems Test (Mini-BESTest), which has been shown to be effective at assessing multiple subdomains of balance in PwP [19]. As secondary motor outcomes, participants completed several other assessments. Balance self-efficacy was assessed using the 16-item Activities Specific Balance Confidence Scale (ABC) [20]. The ABC captures the self-assessed balance confidence of individuals performing tasks of daily living without losing their balance. We have previously shown that ABC correlates with dynamic stability [21]. This measure is reliable, valid, and predictive of fall status and tendency of future falls [22]. Individuals also performed a timed single limb stance task, a balance measure previously shown to improve in response to a progressive resistance exercise intervention [23]. For this task, individuals stood with their hands on their hips while focusing on a single target approximately 2 m in front of them. Individuals were instructed to lift one leg off the ground and avoid hooking their stance limb. Timing started when the foot left the ground and stopped when they touched back down. The outcome for the side/leg most affected by PD was assessed. Mobility was also assessed using the TUG Dual-Task and an additional measure of gait included comfortable walking speed (CWS), which was collected in a similar manner as MWS but individuals were instructed “to walk at a speed that is most comfortable to you”, as previously described [21].

Comprehensive Neurocognitive Battery: Participants were evaluated with a level II neurocognitive battery as defined by the Movement Disorders Society Task Force for diagnosis of PD-mild cognitive impairment (MCI), which included at least two tests in at least five cognitive domains [8]. Cognitive assessments were completed in the ON medication state at the time of day when participants felt their medications were most effective. This battery included the following tests: Attention/Working Memory Domain: 1) Wechsler Adult Intelligence Scale IV (WAIS-IV) letter-number sequencing subtest and 2) Weschler Memory Scale-III (WMS-III) digit span (forward and backward); Memory Domain: 1) 10–36 Spatial Recall Test (10–36) immediate recall and delayed recall and 2) Hopkins Verbal Learning Test −Revised (HVLT-R) total immediate recall and delayed recall; Executive Function Domain: 1) Trail-making test Trails B minus A (Trails B-A), 2) Delis-Kaplan Executive Function System (D-KEFS) Stroop color-word interference test: Stroop Inhibition, and 3) Stroop Inhibition/Switch; Language Domain: 1) phonemic verbal fluency (Controlled Oral Word Association: COWA-CFL) and 2) Semantic/category fluency (animal naming); Visuospatial Skills Domain: 1) Hooper Visual Organization Test (VOT) and 2) Benton Judgment of Line Orientation (JLO); and Processing Speed Domain: 1) D-KEFS Stroop color naming, 2) Stroop word naming, and 3) Trails A.

#### 1.2.2 Additional assessments

Participants were evaluated with the Movement Disorders Society-Unified Parkinson’s Disease Rating Scale (MDS-UPDRS) [24] in ON medication state to evaluate disease severity. Dopaminergic therapy usage (Levodopa Equivalent Dose: LED) and LED-dopamine agonists (DA) were calculated as previously described [25].

### 1.3 Statistical analyses

Scores for individual cognitive tests were used to calculate a normalized (z-) score based on normative values, which control for age, sex, race, and education as appropriate for each test [[26], [27], [28], [29]]. For each participant, individual test z-scores were averaged within each cognitive domain to obtain a domain score, and domain scores were averaged to determine the Cognitive Composite Score (CCS) as a measure of global cognitive function. CCS was the *primary cognitive outcome measure*. To be conservative, multiple linear regressions were performed for each cognitive domain and the CCS to account for the possible influence of duration of disease (DOD)and MDS-UPDRS Part III. The resulting studentized residuals were used in group contrasts and correlation analyses.

Statistical analyses were performed using JMP Pro 16 (SAS Institute, Inc., Cary, NC). Summary statistics were calculated and tested for normality with the Shapiro-Wilk test. Group comparisons (men versus women) were conducted with independent-samples *t*-tests for normally distributed data and nonparametric tests (Wilcoxon rank sum) for non-normally distributed data. Spearman correlation coefficient ρ was used to examine relationships between locomotor function and CCS or cognitive domains. All statistical tests were two-tailed and p < 0.05 was considered significant. Because one goal of the study was hypothesis generation, we did not apply corrections for multiple comparisons. With a sample size of 60, a two-tailed Spearman correlation with an alpha level of 0.05 will have 80 % power to detect a medium effect size of 0.36 and a sample size of 29 will have 80% power to detect a large effect of 0.50.

## 2 Results

### 2.1 Participant characteristics

There were no significant differences between the 38 men and 22 women (60 participants total) in terms of age, education, race, disease duration, LED, MDS-UPDRS Parts I-IV or Total score (Table 1). Women performed better within the processing speed domain compared to men (Table 2). No other group differences were observed with respect to cognitive performance or locomotor function.

**Table 1:** Demographics and participant characteristics.

	**All participants**	**Males**	**Females**	***t/Z/x^2^***	***p***
N	60	38	22	−-	−-
Age	65.6 ± 7.09	65.5 ± 7.57	65.8 ± 6.34	0.16	0.87
Sex: N (%)					
Male	38 (63.3)	38 (100)	22 (100)	−-	−-
Female	22 (36.7)				
Duration of Disease (years)	5.0 (2.0–8.8)	4.0 (2.0–7.3)	7.0 (1.0–10.0)	0.89	0.37
Education (years)	16.0 (14.0–18.0)	16.0 (14.0–18.0)	15.5 (14.0–17.3)	−0.63	0.53
Race: N (%)					
Caucasian	55 (91.7)	34 (89.5)	21 (95.5)	0.71	0.40
African American	5 (8.3)	4 (10.5)	1 (4.5)		
MDS-UPDRS Part I	9.0 (5.3–11.8)	9.0 (5.0–12.0)	8.5 (6.5–11.8)	0.18	0.86
MDS-UPDRS Part II	10.25 ± 5.55	10.24 ± 5.12	10.27 ± 6.36	0.02	0.98
MDS-UPDRS Part III	30.78 ± 13.46	32.00 ± 13.83	28.68 ± 12.83	−0.94	0.35
MDS-UPDRS Part IV	3.0 (0.0–5.0)	3.0 (0.0–5.0)	3.0 (0.0–5.3)	−0.14	0.89
MDS-UPDRS Total	53.37 ± 19.23	54.27 ± 18.58	51.86 ± 20.65	−0.45	0.66
LED	600 (317.5–843.8)	640.6 (290.0–909.1)	575.0 (447.5–757.5)	−0.03	0.98
LED DA	40 (0–240)	7.5 (0–250)	100 (0–232.5)	0.17	0.68
Participants on DA:N (%)	33 (55.9)	20 (52.6)	13 (61.9)	0.48	0.49
De novo participants: N (%)	3 (5.0)	3 (5.0)	0 (0)	2.81	0.09

Mean ± SD reported for normally distributed date, Median (IQR) reported for non-normally distributed variables.

LED: levodopa equivalent dose; LED DA: levodopa equivalent dose for dopamine agonists; MDS-UPDRS: Movement Disorders Society-Unified Parkinson’s Disease Rating Scale.

**Table 2:** Cognitive and Motor Outcome Differences between Men and Women.

	**All participants**	**Males**	**Females**	***t/Z***	***p***
**N**	60	38	22		
**Primary Outcomes**					
Cognitive Composite Score	−0.006 ± 1.01	−0.16 ± 1.08	0.26 ± 0.84	−1.66	0.10
Mini-Best	22.50 (17.25–24.75)	23.0 (18.5–25.0)	21.0 (17.0–23.3)	−1.12	0.26
TUG	9.03 (7.58–10.67)	9.14 (7.5–10.8)	8.97 (7.4–10.3)	−0.31	0.75
MWS	1.60 ± 0.32	1.64 ± 0.33	1.52 ± 0.28	−1.46	0.15
**Secondary Outcomes**					
Attention / Working Memory Domain	−0.003 ± 1.00ǂ	0.14 ± 1.05*	−0.25 ± .90^♦^	−1.42	0.16
Memory Domain	−0.004 ± 1.01	−0.14 ± 1.02	0.23 ± 0.94^+^	1.38	0.18
Executive function Domain	−0.004 ± 1.01†	−0.17 ± 1.12*	0.27 ± .74^+^	−1.73	0.09
Language Domain	−0.004 ± 1.01	−0.14 ± 0.93	0.23 ± 1.11^+^	1.28	0.21
Visuospatial Domain	−0.006 ± 1.01	−0.12 ± 1.06	0.18 ± 0.92	1.08	0.29
Processing Speed Domain	−0.004 ± 1.01	−0.27 ± 0.86	0.43 ± 1.11	**2.39**	**0.02**
ABC	81.0 (70.25–91.0)	82.0 (70.8–92.3)	76.5 (66.5–89.5)	−1.08	0.28
CWS	1.19 ± 0.24	1.20 ± 0.25	1.17 ± 0.22	−0.45	0.65
TUG Dual Task	12.34 (10.02–14.70)	12.2 (9.6–14.0)	12.5 (10.7–15.6)	−0.06	0.95
Most affected single leg stance^#^	5.67 (3.56–15.93)	5.5 (2.9–14.9)**	6.1 (4.8–19.2)	0.88	0.38

Mean ± SD reported for normally distributed date, Median (IQR) reported for non-normally distributed variables.

CCS and cognitive domains adjusted for duration of disease and MDS-UPDRS Part III.

ǂ N = 52, †N = 53, ^#^N = 58, *N = 33, ^+^N = 20, **N = 36, ^♦^N = 19; Bold indicates significant difference.

### 2.2 Correlation between cognition and locomotor outcomes

**Fig. 1:**
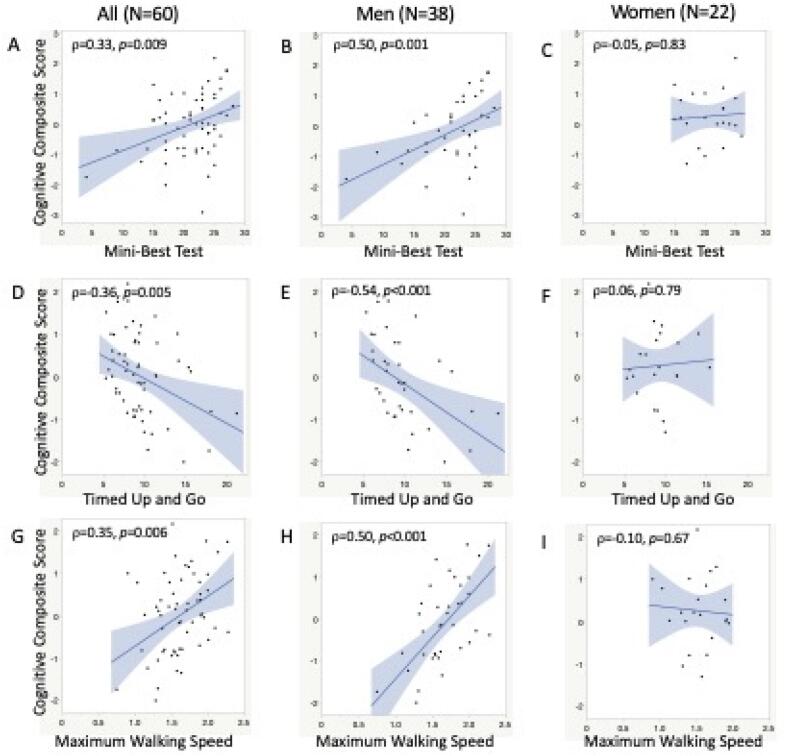
Correlations between primary outcomes. A-C: Correlation of Mini-Best Test with Cognitive Composite score in full group (A), men (B), and women (C). D-E: Correlation of Timed Up and Go with Cognitive Composite score in full group (D), men (E), and women (F). G-I: Correlation of Maximum Walking Speed with Cognitive Composite score in full group (G), men (H), and women (I). CCS adjusted for duration of disease and MDS-UPDRS Part III.

The CCS (primary cognitive outcome) was correlated with all primary motor outcomes for the overall group and men (only), whereas similar relationships were not observed among women (Table 3, Fig. 1). Specifically, positive relationships were observed between the CCS and Mini-BEST Test (balance) for the overall group (*p* = 0.009) and men (*p* = 0.001), as well as between CCS and MWS (gait) for the overall group (*p* = 0.006) and men (*p* < 0.0001) indicating that better balance and gait function are associated with better cognitive performance, particularly in men. There was a negative correlation between CCS and TUG (mobility) for the overall group (*p* = 0.005) and for the men (*p* < 0.001), indicating that better mobility was associated with better cognitive performance. No relationships were observed for women between the CCS and Mini-BEST (*p* = 0.83), MWS (*p* = 0.67), or TUG (*p* = 0.79).

**Table 3:** Spearman Correlations between cognitive performance and primary locomotor outcomes.

	**All participants (N = 60)**			**Men Only (N = 38)**			**Women Only (N = 22)**		
	**Mini-BESTest**	**TUG**	**MWS**	**Mini-BESTest**	**TUG**	**MWS**	**Mini-BESTest**	**TUG**	**MWS**
**Cognitive Composite Score**	0.33**	−0.36**	0.35**	0.50**	−0.54**	0.60**	−0.05	0.06	−0.10
**Attention/Working Memory Domain**	0.15	−0.07	0.17	0.32	−0.24	0.26	−0.33	0.30	−0.25
**Memory Domain**	0.22	−0.32*	0.26	0.33	−0.51**	0.49**	0.08	0.05	−0.10
**Executive Function Domain**	0.31*	−0.36**	0.36**	0.32	−0.39*	0.50**	0.34	−0.23	0.14
**Language Domain**	0.21	−0.22	0.14	0.33	−0.49**	0.48**	0.08	0.15	−0.40
**Visuospatial Skills Domain**	0.28*	−0.35*	0.39**	0.41*	−0.48**	0.54**	0.03	−0.13	0.14
**Processing Speed Domain**	0.30*	−0.39**	0.30*	0.53**	−0.47**	0.49**	−0.03	−0.24	0.15

CCS and cognitive domains adjusted for duration of disease and MDS-UPDRS Part III.

*p < 0.05, **p < 0.01; Bold indicates significant correlation

MWS: maximum walking speed; TUG: timed up and go.

To explore the consistency of our results, we examined the relationships between cognitive performance and our remaining mobility, gait, and balance measures (Table 4: all participants and Tables 5: men and 6: women). The CCS was correlated with several additional measures of balance. Specifically, there were positive relationships between the CCS and ABC (overall group: *p* = 0.003, men *p* < 0.001), and the most affected single leg stance (group: *p* < 0.001, men: *p* < 0.001). There was no relationship between CCS and CWS for the overall group (*p* = 0.17), however a significant correlation was observed among the men (*p* = 0.03) for this additional measure of gait. The CCS was also correlated with our additional measure of mobility, the TUG Dual-Task (overall group: *p* = 0.002, men *p* = <0.001). Similar to primary outcomes, no relationships were observed in women between CCS and these additional measures of mobility, gait, and balance (Table 6). However, the TUG Dual-Task (secondary outcome) was significantly negatively correlated with the executive function (*p* = 0.01) and processing speed (*p* = 0.02) domains in women.

**Table 4:** Spearman correlations between cognitive performance and secondary motor outcomes for all participants.

	**All participants (N = 60)**			
	**ABC**	**CWS**	**TUG Dual Task**	**Most Affected side single leg stance**
**Cognitive Composite Score**	0.38**	0.18	−0.40**	0.43**
**Attention/Working Memory Domain**	0.24	0.01	−0.31*	0.13
**Memory Domain**	0.42**	0.09	−0.36**	0.38**
**Executive Function Domain**	0.35**	0.25	−0.43**	0.44**
**Language Domain**	0.02	0.06	−0.17	0.21
**Visuospatial Skills Domain**	0.39**	0.18	−0.28*	0.42**
**Processing Speed Domain**	0.27*	0.32*	−0.47**	0.28*

CCS and cognitive domains adjusted for duration of disease and MDS-UPDRS Part III.

*p < 0.05, **p < 0.01; Bold indicates significant correlation

**Table 5:** Spearman correlations between cognitive performance and secondary locomotor outcomes in men.

	**Men (N = 38)**			
	**ABC**	**CWS**	**TUG Dual Task**	**Most Affected side single leg stance**
**Cognitive Composite Score**	0.60**	0.35*	−0.52**	0.56**
**Attention/Working Memory Domain**	0.34	0.09	−0.38*	0.36*
**Memory Domain**	0.59**	0.26	−0.52**	0.53**
**Executive Function Domain**	0.36*	0.31	−0.38*	0.46**
**Language Domain**	0.35*	0.30	−0.46**	0.32
**Visuospatial Skills Domain**	0.51**	0.26	−0.41*	0.52**
**Processing Speed Domain**	0.38*	0.47**	−0.51**	0.46**

CCS and cognitive domains adjusted for duration of disease and MDS-UPDRS Part III.

*p < 0.05, **p < 0.01; Bold indicates significant correlation

**Table 6:** Spearman correlations between cognitive performance and secondary locomotor outcomes in women.

	**Women (N = 22)**			
	**ABC**	**CWS**	**TUG Dual Task**	**Most Affected side single leg stance**
**Cognitive Composite Score**	0.03	−0.14	−0.18	0.02
**Attention/Working Memory Domain**	0.09	−0.30	−0.09	−0.16
**Memory Domain**	0.27	−0.13	−0.14	−0.02
**Executive Function Domain**	0.31	0.15	−0.55*	0.17
**Language Domain**	−0.43	−0.30	0.24	0.05
**Visuospatial Skills Domain**	0.28	0.02	−0.05	0.18
**Processing Speed Domain**	0.08	0.12	−0.52*	−0.11

CCS and cognitive domains adjusted for duration of disease and MDS-UPDRS Part III.

*p < 0.05, **p < 0.01; Bold indicates significant correlation

### 2.3 Exploratory analyses of cognitive function

While the CCS was the primary outcome for this study, to guide planning of future work, we explored which domains were driving the relationships between CCS and motor function. This exploratory analysis revealed a similar pattern such that the correlations within the overall group were driven by the men. Specifically, the Mini-BEST Test was correlated with the visuospatial skills (group: *p* = 0.04, men: *p* = 0.015) and processing speed (group: *p* = 0.03, men: *p* = 0.001) domains. The TUG was negatively correlated with the executive function (group: *p* = 0.007, men: *p* = 0.03), memory (group: *p* = 0.02, men: *p* = 0.002), visuospatial (group: *p* = 0.01, men: *p* = 0.004), and processing speed domains (group: *p* = 0.004, *p* = 0.006). MWS was associated with the executive function (group: *p* = 0.008, men: *p* = 0.003), visuospatial skills (group: *p* = 0.003, men: *p* = 0.001), and processing speed (group: *p* = 0.03, men: *p* = 0.004) domains. For the entire group, the Mini-Best test was correlated with executive function (group: *p* = 0.02), without significant correlation in men. No significant correlations between the primary motor function outcomes and cognitive domains were observed among the women.

Unique relationships among men. Additional unique relationships were observed for the men (only) such that the TUG was negatively correlated with the language domain (*p* = 0.003) and MWS was correlated with the memory (*p* = 0.004) and language (*p* = 0.004) domains. No discrete relationships were observed among the women.

## 3 Discussion

Overall, our investigation reveals a significant association of global cognitive performance with balance, mobility, and gait among PwP. Notably, a novel aspect of our study lies in the use of a comprehensive neurocognitive battery, which reveals that the relationships between cognitive performance and measures of balance, mobility, and gait seems to be most influenced by performance in domains of executive function, visuospatial skills, and processing speed, with an additional influence of memory on the relationship with mobility. Moreover, upon stratifying the sample by sex, we observed that the relationships between locomotor function and cognitive performance were discernible solely among male participants. Specifically, correlations between measures of gait and mobility with global cognition, memory, executive function, language, visuospatial skills, and processing speed were evident exclusively in men, and balance was correlated with global cognition, visuospatial skills, and processing speed only in men. Importantly, these findings suggest that cognitive performance in multiple domains might serve as an indicator of susceptibility to gait, mobility, and balance dysfunction in male PwP. Interestingly, the only relationships between locomotor function and cognition in women was between TUG dual task performance (mobility) and executive function and processing speed. From a clinical perspective, the detection of cognitive impairment should prompt consideration of rehabilitation interventions targeting gait, mobility, and balance in PwP.

The current study showed relationships between gait/balance/mobility and executive function as well as processing speed, key domains for dual-task performance, among PwP. This finding extends prior work in the areas of PD and cognitive impairment [2,9,[30], [31], [32], [33]]. PwP show significant declines in walking speed, stride length, and stride frequency under dual-task conditions, which suggests that the effects of dual-tasking in PwP emphasizes the consequences of cognitive load on gait in PwP [2,30]. Furthermore, attributes of gait have been correlated with executive function (working memory and decision making) and other cognitive processes such as attention and problem solving [9,31,32]. The mechanisms underlying the relationships between cognition and locomotor function are not completely understood, but are likely related to neurodegeneration or alteration of brain structures important for both gait and cognition [34]. For example, changes in brain iron accumulation occur in PD and have been associated with both motor function and cognition [35], and there are differences in this accumulation between men and women [36]. Further, alterations of frontal-striatal circuits, executive-attention networks, and visuospatial circuits, among others, can negatively impact both locomotor function and cognition [34].

Balance instability is a common symptom in PwP and the mini-BESTest is a validated tool to assess balance in this population [19]. This instability has also been observed in individuals with MCI, with studies indicating a correlation between cognitive decline and static postural balance [33]. Moreover, among various types of cognitive impairment (subjective cognitive impairment, MCI, Alzheimer's dementia), balance control is correlated with executive function such that concomitantly, all aspects of balance control decline as cognitive impairment worsens [37]. This relationship between executive function and balance control is also observed among PwP. One study showed that executive function was correlated with dynamic postural balance (i.e. balance while the body is in motion) in PwP [38]. Poorer performance on tests of executive function were correlated with postural instability (balance), and this finding was supported by the findings of multiple previous studies which correlated executive function to balance impairments/postural instability [[39], [40], [41], [42]]. In summary, the relationship we observed between balance and executive function in PwP has been corroborated by multiple previous studies.

Furthermore, we found a relationship between processing speed and mobility (TUG) among PwP. This is consistent with a study that showed reduced processing speed was associated with impaired turning ability (turn duration and number of steps to turn) in PwP [1]. Taken together, these data suggest processing speed plays a key role in performing complex mobile tasks (i.e. standing and turning) in PwP. Furthermore, prior work found a relationship between attentionally demanding cognitive tasks and TUG in PwP, suggesting a relationship between mobility and attention in PwP [43]. Two studies found relationships between TUG performance and executive function in dual-task performance [44,45]. This relationship between mobility and executive function, when combined with the relationship between TUG and processing speed/attention, suggests a connection between executive function, processing speed/attention, and dual-task performance in PwP. Future research directions could focus on conducting longitudinal studies to track cognitive and locomotor decline over time in PD, particularly investigating how cognitive impairments predict or exacerbate locomotor symptoms.

PD exhibits a higher prevalence among men than women [[46], [47], [48], [49]]. Specifically, twice as many men develop PD than women [47,48]. However, women demonstrate a more rapid disease progression and higher mortality rate compared to men [49]. Moreover, sex differences are associated with distinct PD phenotypes [46,[50], [51], [52], [53], [54], [55]]. Men have a heightened risk of developing camptocormia [50] and freezing of gait, the most debilitating motor complication of PD during its progression [51]. Additionally, falling is listed among the predictors of disease progression in women [52]. PD typically manifests in women with a milder phenotype, although defining this phenotype remains challenging [53,54]. Motor symptoms in women tend to manifest later than in men, with reduced rigidity, tremors more frequently presenting as the initial symptom, and a heightened propensity for developing postural instability and increased risk of levodopa-related motor complications [46]. Notably, one study indicated that women perform better on the motor section of the UPDRS compared to men, yet exhibit a significantly higher prevalence of dyskinesias [55]. Consequently, men may experience motor impairment sooner and more frequently than women with PD.

Our investigation confirmed these trends as, when stratified by sex, relationships between locomotor function and cognitive performance were observed solely among men. Multiple studies have indicated that men with PD are more susceptible to cognitive impairment (MCI, dementia) compared to women [[56], [57], [58], [59]]. In fact, one study identified male sex as the primary predictor in transitioning from no cognitive impairment to either PD-MCI or PD dementia (PDD) [60]. Specifically, in the current study, only men demonstrated correlations between measures of gait and mobility and global cognition and most cognitive domains. These findings suggest that cognitive performance, particularly in domains of executive function, processing speed, and visuospatial skills, may serve as a marker of risk for gait and balance dysfunction in men with PD. In contrast, with the exception of relationships between TUG dual-task and executive function/processing speed performance, no relationships between cognitive performance and locomotor function were observed among women with PD. This lack of association could be related to fewer women in the sample having cognitive impairment, thus introducing ceiling effects, or may be related to differential neurobiological relationships between cognition and locomotor function in women. Another consideration is that the smaller sample of women may have introduced a type II error in the current study. However, the magnitude of the Spearman correlation values in women did not approach what was seen among men, so this seems less likely. Further exploration of neurobiological mechanisms connecting cognitive functions to locomotor impairments in larger samples of men and women with PD is crucial, as such research endeavors can inform the development of neuroprotective interventions aimed at preserving cognitive and locomotor function in individuals with PD.

To our knowledge, this study is the first to comprehensively investigate gait, balance, and mobility separately in relation to cognitive performance in PD, using a level II comprehensive neurocognitive battery as recommended by the Movement Disorders Society Task Force [8]. While previous studies have examined locomotor-cognition relationships in PD, none employed a level II neurocognitive battery [[61], [62], [63]]. For example, PD-MCI has been shown to be associated with postural instability and gait disorder (PIGD) [61]. In another previous study, correlations between motor function and specific cognitive domains have been made through the use of the UPDRS Parts II and III, Montreal Cognitive Assessment (MoCA), and the Frontal Assessment Battery (FAB) in PwP [62]. A third previous study correlated cognitive and motor performance during dual-tasking in PwP [63]. Furthermore, our study utilized clinically proven assessment tools for each locomotor function domain, all recommended by the Movement Disorders Society Rating Scales Committee [64]. Specifically, we employed MWS for gait assessment, the Mini-BESTest for balance evaluation, and the TUG test for mobility assessment. These assessments offer reliable and valid measures for evaluating motor functionality in PwP [18,[65], [66], [67], [68], [69]]. Our study thus adds valuable insights into the relationship between locomotor function and cognition in PD, enhancing our understanding of this complex interplay.

Limitations of the study include the observational, cross-sectional, and correlational nature of this study. Although the findings are compelling and fit within our understanding of the relationships between locomotor function and cognitive performance involved in PwP, future work will need to include investigation of these associations over time and in response to physical rehabilitation interventions. Techniques are available for such studies and can be further explored based on the current study [70,71]. These findings should also be replicated in a larger sample of women, specifically. Another limitation is that this study did not address some potential confounders that could influence cognition and locomotor function, such as sleep quality, physical activity levels, and mood. Further, we report several hypothesis-generating exploratory analyses and did not correct for multiple comparisons. However, the focus of the current study is the association of independent measures of mobility, gait, and balance in relation to global cognitive function (CCS). Finally, we did not include a non-PD control group. Although this would have been informative, the focus of this study was to identify the association between locomotor function (specifically, mobility, gait, and balance) and global cognitive performance in PD, but these relationships among healthy adults without motor impairment should be explored in future investigations.

## 4 Conclusions

In conclusion, our study adds to the body of knowledge regarding the relationship between global cognitive performance and locomotor function in PwP. We found that relationships between locomotor function and cognitive performance were evident only among men. Specifically, only men showed correlations between measures of gait, balance, and mobility and global cognition, executive function, visuospatial function, and processing speed. Future longitudinal investigations are needed to unravel the temporal association between cognitive decline and alterations in gait, balance, and mobility, shedding light on disease progression and identifying potential biomarkers or predictors for clinical management strategies. Additionally, exploring the underlying mechanisms linking cognition and locomotor function may inform the development of targeted interventions to preserve cognitive and locomotor abilities in PD. This study highlights the potential impacts of PD on cognitive and locomotor function, especially among men.

## 5 Disclosures

### 5.1 Financial Support

This study was funded by the NIH (AWA: K23NS080912, KHW: 1 T32 HD071866), UAB Center for Exercise Medicine, Parkinson’s Disease Foundation, and the Rehabilitation Research Resource to Enhance Clinical Trials (REACT).

### 5.2 Ethical Compliance Statement

This study was approved by the Institutional Review Board at the University of Alabama at Birmingham and written informed consent was obtained from all participants. We confirm that we have read the Journal’s position on issues involved in ethical publication and affirm that this work is consistent with those guidelines.

## CRediT authorship contribution statement

**Amy W. Amara:** Writing – review & editing, Writing – original draft, Visualization, Supervision, Resources, Project administration, Methodology, Investigation, Funding acquisition, Formal analysis, Data curation, Conceptualization. **Kimberly H. Wood:** Writing – review & editing, Writing – original draft, Visualization, Project administration, Methodology, Investigation, Formal analysis. **Aya M. Miften:** Writing – review & editing, Writing – original draft. **Lina Kleinschmidt:** Writing – review & editing. **Corey S. White:** Writing – review & editing, Writing – original draft. **Allen Joop:** Writing – review & editing, Project administration, Investigation, Data curation. **Raima A. Memon:** Writing – review & editing, Project administration, Methodology, Investigation. **Jennifer Pilkington:** Writing – review & editing, Project administration, Methodology, Investigation. **Jutaluk Kongsuk:** Writing – review & editing, Project administration, Investigation, Data curation. **Corina Catiul:** Writing – review & editing, Project administration, Methodology, Investigation. **Adeel A. Memon:** Writing – review & editing, Writing – original draft, Project administration, Investigation. **Marcas M. Bamman:** Writing – review & editing, Writing – original draft, Resources, Funding acquisition, Conceptualization. **Christopher P. Hurt:** Writing – review & editing, Validation, Resources, Methodology, Investigation, Funding acquisition, Data curation, Conceptualization.

## Declaration of competing interest

The authors declare that they have no known competing financial interests or personal relationships that could have appeared to influence the work reported in this paper.
